# Navigating the Unexpected: Dysphagia Lusoria Complicates Severe Pneumonia With Parapneumonic Effusion

**DOI:** 10.7759/cureus.67812

**Published:** 2024-08-26

**Authors:** Pyae Kyaw, Nava R Sharma, Khin Soe, Yu Shia Lin, Monica Ghitan, Shaurya Sharma

**Affiliations:** 1 Internal Medicine, Maimonides Medical Center, Brooklyn, USA; 2 Infectious Disease, Maimonides Medical Center, Brooklyn, USA

**Keywords:** dysphagia lusoria, pneumonia, aberrant left subclavian artery (alsa), parapneumonic effusions, dysphagia

## Abstract

Managing pneumonia, especially when complicated by underlying anatomical anomalies, presents unique challenges that require a nuanced and multidisciplinary approach. Dysphagia lusoria, a rare vascular anomaly where the right subclavian artery originates aberrantly, can coexist with other thoracic conditions, complicating both diagnosis and treatment. Understanding the interplay between such anomalies and common infections like pneumonia is crucial for optimal patient outcomes. This case report describes a 33-year-old male with a history of recurrent pneumonia in the past who presented to the emergency department (ED) with right flank pain and dyspnea persisting for one week. Initial investigations revealed moderate parapneumonic pleural effusion and right lower lobe pneumonia. At the time, an aberrant origin of the right subclavian artery (ARSA) (dysphagia lusoria) was incidentally detected on imaging. The patient’s management included antibiotic therapy tailored for pneumonia and the placement of a chest tube for pleural effusion drainage. Despite intermittent dysphagia, surgical intervention for dysphagia lusoria was deferred due to its minimal impact on daily functioning. The patient improved significantly with supportive care and antibiotics, highlighting the complexity of managing pneumonia complicated by anatomical anomalies. This case underscores the importance of multidisciplinary management and tailored treatment strategies in addressing intricate clinical scenarios.

## Introduction

Dysphagia lusoria, characterized by an aberrant origin of the right subclavian artery (ARSA), is a rare congenital anomaly resulting from developmental variations in the aortic arch during embryogenesis. Its reported incidences range from 0.5% to 1.8% in the adult population [1,2]. Typically diagnosed in adulthood, dysphagia lusoria presents with symptoms related to esophageal compression by the anomalous vessel, though clinical manifestations can vary widely [1,3]. We present the case of a 33-year-old male with a history of recurrent pneumonia who presented with respiratory symptoms and was incidentally diagnosed with dysphagia lusoria during evaluation for parapneumonic pleural effusion. This case highlights the diagnostic complexities and the multidisciplinary approach necessary for managing patients with concurrent respiratory conditions and anatomical anomalies like dysphagia lusoria.

## Case presentation

A 33-year-old male presented to the emergency department (ED) for the third time within a week, complaining of right flank pain and shortness of breath, persisting for one week. He had a productive cough and intermittent fever ranging from 100 to 101°F for the past two weeks. The fever was associated with shortness of breath and severe stabbing pain in the right chest and flank areas, rated 8 out of 10, and exacerbated by coughing. The patient had been evaluated twice in the ED earlier that week for abdominal pain. He was treated with symptomatic management for possible renal stones during those visits.

His significant past medical history included pneumonia during his teenage years and intermittent mild difficulty swallowing, particularly with dry foods, which used to be relieved by drinking soda. He denies any diagnosis of immunodeficiency. Denies any recent h/o dental procedure.

On examination, the patient appeared to be in mild respiratory distress with a temperature of 37.2°C, an elevated heart rate of 110 bpm, blood pressure of 129/79 mmHg, an elevated respiratory rate of 25 breaths per minute, and an oxygen saturation (SpO2) of 94% on room air. Auscultation of the chest revealed reduced breath sounds in the right lower chest. The cardiac examination was unremarkable, with normal heart sounds and no murmurs. Abdominal examination showed tenderness in the right upper quadrant without rebound or guarding, along with right costovertebral angle tenderness. No lower extremity edema was noted. The patient’s oral cavity, including the teeth, gums, and other structures, appeared unremarkable.

**Figure 1:**
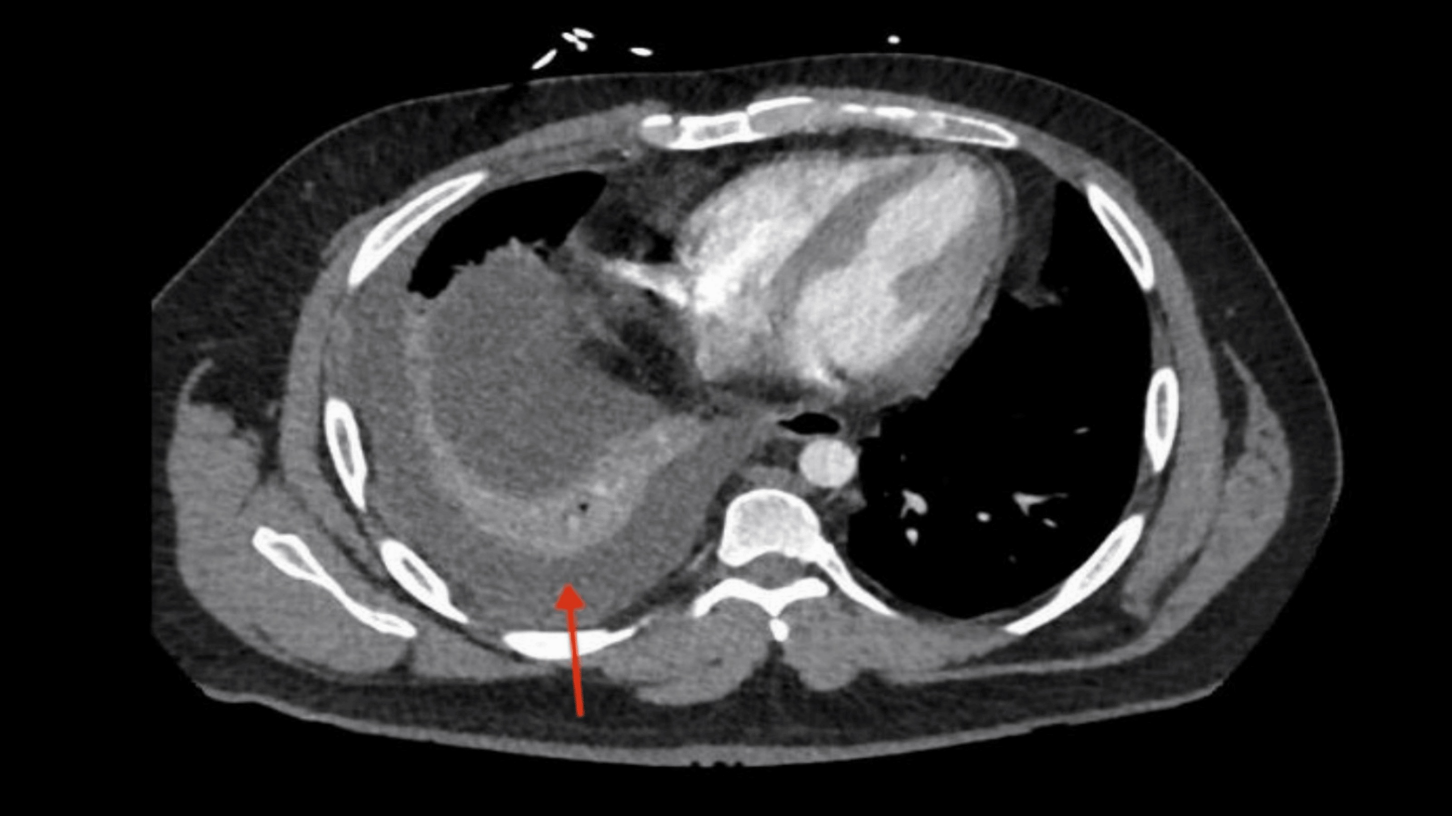
Right basal pleural effusion shown by the red arrow

**Figure 2:**
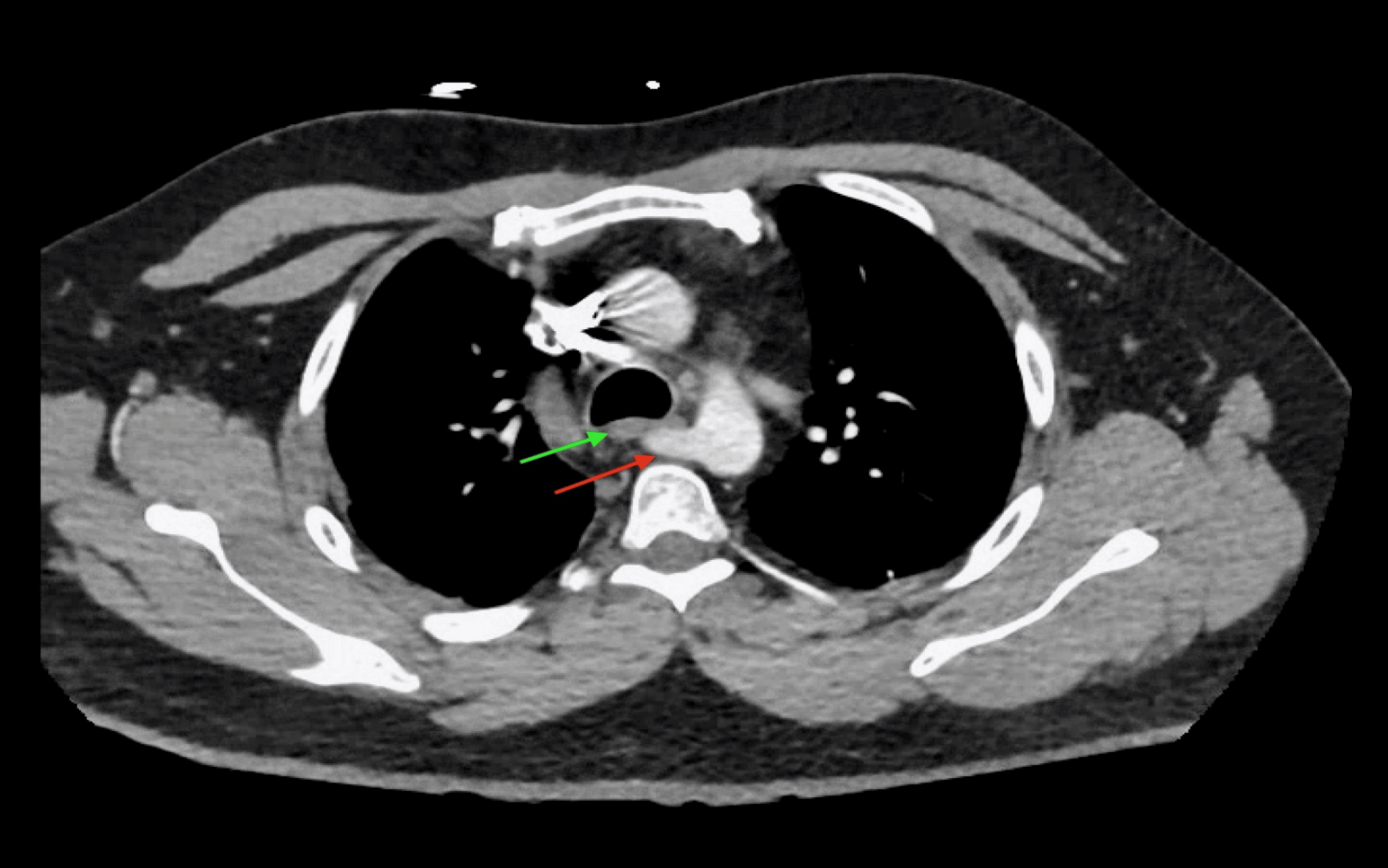
The green arrow indicates the esophagus, while the red arrow indicates the aberrant origin of the subclavian artery from the aorta

Laboratory findings revealed leukocytosis with a white blood cell (WBC) count of 19 × 10^9^/L, while other parameters were within normal limits. CT chest with contrast revealed no pulmonary embolism but noted moderate parapneumonic effusion without separation and right lower lobe pneumonia, as shown in Figure 1. An ARSA was also observed, as shown in Figure 2.

The patient was started on intravenous antibiotics (cefepime, metronidazole, and doxycycline). A septic workup was initiated, and consultations with pulmonary and infectious disease (ID) specialists were made. A chest tube was placed, and pleural fluid analysis showed low glucose, elevated lactate dehydrogenase (LDH), and high WBC with neutrophilic predominance consistent with exudative pleural effusion, as shown in Table 1. Pleural fluid gram stain and culture were negative.

**Table 1:** Pleural fluid analysis Pleural fluid analysis showed low glucose, elevated LDH, and high WBC with neutrophilic predominance. LDH, lactate dehydrogenase; WBC, white blood cell

Parameter	Patient's value	Normal reference range
Albumin (fluid)	2.8 g/dL	1.0-2.5 g/dL
Amylase (fluid)	22 U/L	<160 U/L
Glucose (fluid)	12 mg/dL	60-100 mg/dL
LDH (fluid)	1820 U/L	<200 U/L
Specific gravity (fluid)	1.02	1.005-1.030
Total protein (fluid)	5.0 g/dL	0.2-1.5 g/dL
Fluid color	Yellow	Clear to pale yellow
Fluid appearance	Cloudy	Clear to slightly cloudy
Fluid WBC	3372 cells/μL	<300 cells/μL
Fluid RBC	5000 cells/μL	<1000 cells/μL
Fluid neutrophil (%)	79%	0-25%
Fluid lymphocyte (%)	17%	50-70%
Fluid monocytes (%)	2%	0-8%
Fluid eosinophil (%)	2%	0-6%

An echocardiogram showed no vegetation. A barium swallow study was performed due to a history of intermittent dysphagia and revealed proximal bolus retention, retroflow below the upper esophageal sphincter, and asymmetrical flow with distal esophageal retentions, as shown in Video 1. The thoracic surgery was consulted, who determined that emergent surgical intervention for dysphagia lusoria is unnecessary and recommended implementing dietary modification. The only potential predisposing factor identified was dysphagia lusoria, potentially leading to silent aspiration leading to pneumonia. Given the absence of other clear sources of infection, the incidental finding of dysphagia lusoria was considered a predisposing factor.

**Video 1:** Video showing barium swallow study in a patient with dysphagia lusoria Barium swallow studies revealed proximal bolus retention, retroflow below the upper esophageal sphincter, and asymmetrical flow with distal esophageal retentions.

The patient improved significantly after a few days of inpatient admission. His WBC count trended down, he was afebrile, and his pain was managed effectively. The chest tube was removed, and a follow-up chest X-ray showed no pneumothorax. He was discharged after 13 days of admission with a prescription for oral levofloxacin to continue for 13 days.

At the post-discharge follow-up, the patient reported feeling well and returning to his baseline activity level without any significant respiratory or gastrointestinal symptoms. He was advised to continue following up with his primary care provider and thoracic surgery team for ongoing evaluation and management.

## Discussion

This case report describes a 33-year-old male presenting with recurrent pneumonia complicated by a parapneumonic effusion, which contributed to the newly diagnosed dysphagia lusoria, an anatomical variant of the right subclavian artery. Dysphagia lusoria is a condition caused by an ARSA, which is a rare variant resulting from the embryonic development of the aortic arch, occurring with an incidence ranging from 0.5% to 1.8% of the adult population [1]. It occurs due to the persistence of the seventh intersegmental artery and the involution of the fourth vascular arch, leading to the formation of the right dorsal aorta [2]. Typically, the aberrant artery passes behind the esophagus, although in a small percentage of cases, it may pass in front of it [3].

Up to 60-80% of individuals with this condition experience no symptoms throughout their lives [1,4]. When symptoms do occur in adults, they typically manifest as dysphagia due to mechanical obstruction. Symptoms often include difficulty swallowing solids, regurgitation of unchewed food, post-meal bloating, chest pain, and symptoms that vary with body position [1]. Additional complaints may involve coughing, chest pain, or manifestations like Horner’s syndrome [1,4]. In rare instances, patients may present with complications, such as aneurysmal rupture of the aberrant artery or Kommerell’s diverticulum [5]. The presentation was consistent with the imaging findings, despite the anatomical anomaly, as the patient primarily complained of respiratory issues, including significant right flank pain, shortness of breath, and chest discomfort that worsened with coughing.

The physical examination typically shows no abnormalities, although occasionally asymmetrical radial pulses may be detected, particularly in the presence of other vascular anomalies [1]. Upper GI endoscopy usually appears normal, but in some cases, there may be a pulsatile extrinsic compression noted on the posterior wall of the esophagus [6]. Esophageal manometry often reveals non-specific findings, such as a high-pressure zone where the aberrant vessel compresses the esophagus, along with elevated peristaltic pressures proximal to the compression site [7]. Barium esophagogram is the preferred initial diagnostic modality, demonstrating an oblique ascending extrinsic compression above the level of the aortic arch, often accentuated by administering a solid bolus to provoke symptoms [8]. Imaging techniques such as CT angiography or MRI angiography have largely replaced conventional angiography, offering detailed anatomical visualization of the aortic arch and surrounding structures, as well as detecting any concurrent intrathoracic pathologies [9]. These advanced imaging modalities, including 3D reconstructions, provide a comprehensive assessment for accurate diagnosis in patients presenting with dysphagia.

Management of dysphagia lusoria hinges on the severity of symptoms. For mild to moderate cases, symptomatic treatment involves lifestyle adjustments, such as dietary modifications, slower eating, thorough chewing, smaller bites, and consuming liquids with meals [1,10]. Acid suppression and prokinetic agents like proton pump inhibitors can also be used, as demonstrated in studies where these medications alleviated symptoms in some patients [10]. Whether dysphagia in these cases is primarily due to gastroesophageal reflux or a motility disorder remains speculative. When conservative measures fail to provide relief, surgical intervention can be considered [11].

The patient’s presentation with respiratory symptoms alongside intermittent dysphagia suggests a complex clinical picture. Respiratory symptoms could indicate possible compression or irritation of adjacent structures by the aberrant vessel, leading to conditions such as tracheal compression or irritation of the recurrent laryngeal nerve. Intermittent dysphagia, particularly with dry foods, and its relief with soda intake might suggest mechanical obstruction or irritation of the esophagus due to the aberrant artery’s anatomical position. These symptoms together highlight the varied clinical manifestations that can arise from the presence of an aberrant right subclavian artery, underscoring the importance of a thorough history taking, diagnostic evaluation, and tailored management approach. Furthermore, the association with microaspiration secondary to dysphagia raises concern for potential complications, such as aspiration pneumonia with parapneumonic effusion.

In our patient, initial management focused on addressing acute respiratory symptoms and sepsis, including antibiotic therapy tailored to cover likely pathogens causing pneumonia and pleural effusion. The interdisciplinary approach involving pulmonary specialists, ID consultants, and interventional radiologists for chest tube placement exemplified the comprehensive care required in managing such complex cases. The decision not to pursue surgical intervention for dysphagia lusoria was based on the absence of significant clinical impact on the patient’s quality of life or daily functioning. Instead, emphasis was placed on supportive measures and outpatient follow-up to monitor for any potential long-term implications or complications related to the anatomical anomaly.

## Conclusions

This case emphasizes the critical role of dysphagia lusoria, an anatomical anomaly, as a potential underlying cause of pneumonia. The 33-year-old male patient, who had no other comorbidities, presented with significant right-sided pneumonia and parapneumonic effusion. The only notable predisposing factor identified was dysphagia lusoria, where an aberrant right subclavian artery compresses the esophagus, potentially leading to silent aspiration and subsequent pneumonia. In cases where pneumonia and parapneumonic effusion arise without a clear etiology, dysphagia lusoria should be actively considered as a contributing factor. This case highlights the necessity of including anatomical anomalies in the differential diagnosis, particularly in young patients with unexplained respiratory conditions, to ensure appropriate management and treatment.
